# Reference Birth Weight, Length, Chest Circumference, and Head Circumference by Gestational Age in Japanese Twins

**DOI:** 10.2188/jea.13.333

**Published:** 2007-11-30

**Authors:** Syuichi Ooki, Yoshie Yokoyama

**Affiliations:** 1 Department of Health Science, Ishikawa Prefectural Nursing University.; 2 School of Health Sciences, Faculty of Medicine, Kyoto University.

**Keywords:** twins, intrauterine growth, birth weight, reference standards, gestational age

## Abstract

BACKGROUND: Numerous birth weight standards for twins have been reported in western countries, whereas little is in Japan. The aim of this study is to present birth weight, birth length, chest circumference, and head circumference references, clarifying features related to these body size parameters, and to compare our birth weight references with recent report of birth weight norms of Japanese twins using the vital statistics.

METHODS: The subjects consisted of 1,061 twin pairs in total, with birth years ranging from 1968 through 1990. Data was obtained from the Twins Protocol Questionnaire, which asked for information about twins’ growth and development in infancy, and the “Maternal and Child Health Handbook,” which was presented by Ministry of Health and Welfare. Statistical means, standard deviations, and selected percentiles by gestational age were calculated and smoothed using data that contained at least gestational age and one of the four items.

RESULTS: Birth weight was significantly lighter than that of singletons when three additional parameters, especially chest and head circumference, were not measured. Gestational age was correlated with weight, length, chest circumference, and head circumference, in that order, for both sexes. Compared with singletons, birth weight difference in twins was marked and slight difference was observed as to length, whereas no difference was observed as to chest and head circumference. The present results as to birth weight were consistently similar to the birth weight norms of twins using vital statistics in Japan.

CONCLUSION: Growth standards for twins, especially as to birth weight, are essential to understand and evaluate intrauterine growth of twins.

In Japan, like as in other developed countries, twinning rates have been increasing;^1^ here, however, little information is available concerning twins’ growth, partly because there is no population-based twin registry, making it very difficult to collect growth data of twins at birth.

Twins in Japan are known to be several times more prone to die perinatally than singletons.^2^ The increased risk of adverse outcomes in twin births is mainly attributed to a markedly greater proportion of very low birth weights. Because birth weight is the strongest indicator of the risk of perinatal death, birth weight norms are important both for clinical practices and for epidemiologic studies.^3^ It is well known that intrauterine growth of twins differs from that of singletons. Many studies concerning birth weight standards by gestational age have been reported.^3^^-^^14^ Other body size parameters at birth, such as birth length,^6^^,^^9^ chest circumference,^6^^,^^9^ and head circumference^6^^,^^9^^,^^12^ have not been reported as consistently as birth weight, partly because these parameters are not routinely used as indicators of perinatal risk. Comparing these characteristics in twins with those of singletons may provide clues about the patterns of twin growth.

Previous studies have been conducted mainly in western countries. In Japan, relevant factors, such as the perinatal medical system, twins’ birth rates, and body size of mothers, differ significantly from western countries. Recently, twin birth weight norms in Japan based on birth records in the vital statistics were presented;^14^ these norms are probably the most reliable ones in Japan. The limitations of these norms are that the data is calculated by 100g intervals, and the gestational ages are recorded in weeks, not days. Moreover, no norms are presented regarding birth length, chest circumference and head circumference at birth. The aim of this study is to compare our birth weight data with these norms and to present birth references for other body size parameters using a large-scale sample of normally developed Japanese twins.

## METHODS

### Subjects

The subjects consisted of 1,061 twin pairs (2,122 twins), all of whom were applicants to the secondary education school attached to the faculty of education of the University of Tokyo from 1981 through 2002 (birth year ranged: 1968-1990). This school was established in 1948 and adopted a unique entrance system. Of the approximately 50 pairs of twins, 12 years of age and living in the Tokyo metropolitan area, who take an examination every year, about 15 pairs are admitted. All the parents of applicants must hand in a Twins Protocol Questionnaire, which gathers information on family structure, obstetrical findings of mothers, physical growth, zygosity, and motor and mental development of twins from birth through 11 years of age. One of the parents of each applicant, usually the mother, participates in a medical interview by two or three interviewers (including, from 1988 on, one of the present authors, Ooki), in which their responses to the questionnaire are checked carefully. The data of this study are based on the records from the Twins Protocol Questionnaire and the “Maternal and Child Health Handbook,” presented by the Ministry of Health and Welfare for all pregnant women, including obstetrical records written by obstetricians. The data obtained by these procedures were accepted without modification; therefore, the method and accuracy of measurements could not be ascertained.

Zygosity was determined by means of the questionnaire^15^^,^^16^ and DNA/blood testing. All same-sex twins and their mothers answered the zygosity questionnaire, on the basis of which twins’ zygosity was determined with an accuracy of more than 95%. Zygosity diagnosis using genetic markers or DNA polymorphisms was performed for those twin pairs who were admitted to the school. The characteristics of the subjects are presented in Table 1.

**Table 1.** Basic characteristics of the subjects (2122 twins).

sex	male	997	(47.0%)
	female	1125	(53.0%)

maternal age at twins birth (year) ^a^	means	29.0	
	median	28.0	
	range	19-43	

paternal age at twins birth (year) ^b^	means	31.9	
	median	31.0	
	range	19-53	

interval between marriage and twin birth (year) ^c^	means	3.6	
	median	3.0	
	range	0-19	

use of ovulatory drugs	yes	58	( 2.7%)
	no	1914	(90.2%)
	unknown	150	( 7.1%)

presentation	vertex	1353	(63.8%)
	non-vertex	500	(23.6%)
	unknown	269	(12.7%)

gestational age (day) ^b^	means	265.5	
	median	268	
	range	190-320	

parity	0	1094	(51.6%)
	1	804	(37.9%)
	2	200	( 9.4%)
	3-4	22	( 1.0%)
	unknown	2	( 0.1%)

zygosity	monozygotic	1434	(67.6%)
	same-sex dizygotic	296	(13.9%)
	opposite-sex dizygotic	242	(11.4%)
	unclassified	150	( 7.1%)

neonatal asphyxia	yes	121	( 5.7%)
	no	2001	(94.3%)

a: 8 missing values (4 pairs)

b: 24missing values (12 pairs)

c: 26 missing values (13 pairs)

Birth complications of mothers or twins, for example placenta previa, placental abruption, coiling of the umbilical cord, neonatal asphyxia, growth discordant twins, and twin-to-twin transfusion syndrome, were observed to varying degrees. None of these were grounds for exclusion from the study. In general, it was very difficult to set clear and consistent inclusion/exclusion criteria, as more than ten percent of the present subjects had at least one of the complications mentioned above. Moreover, no subjects showed apparent growth retardation at 11 or 12 years of age. Surely this represents one of the largest and most thorough sets of accurate growth data on twins in Japan, especially because zygosity testing is very rare in Japan. Informed consent concerning statistical analysis of the subjects’ data was performed by written documents as part of the application process.

### Statistical Methods

First, factors that affect body size parameters at birth were confirmed by stepwise regression analysis, with a threshold significance level of 0.10. The variables considered were sex, birth order in twins (first-twin or second-twin), gestational age, maternal and paternal age at twin birth, intervals between marriage and twin birth, birth year of twins, parity, zygosity, and presentation. For qualitative variables, the following codes were used. Sex, female: 0, male:1; Birth order, first-born:1, second-born:0; Zygosity, monozygotic:0, dizygotic:1; Presentation, non-vertex:0, vertex:1.

Next, body size parameters were analyzed according to the missing data conditions; for example, birth weight was analyzed whether or not birth length was measured.

The effect of parity was analyzed according to sex. Next, the correlations of gestational age and body size parameters were analyzed.

Reference body size parameters at birth were analyzed according to sex, considering the results of above mentioned procedures. Means, standard deviations, and selected percentiles by gestational age were calculated. In the process of calculating percentile values, the critical effect of outliers based on birth complications, if they existed, were expected to be excluded.

Smoothing of growth curves was performed by a cubic spline function and compared with previously reported norms for the general population^17^ and twins^14^ in Japan.

Statistical analyses were performed using SAS^®^ for Windows.^18^ Smoothing of growth curves was performed using PROC TRANSREG program, by specifying the ‘pspline’ model.

### Comparison standards

The general population standards (i.e., singletons) that we adopted were based on hospital data presented by a research group of the Ministry of Health and Welfare in 1998.^17^ In this study, standards for birth weight, length, chest circumference, and head circumference by gestational ages from 22 to 41 weeks were determined by the analyses of birth size data on 1,133 infants whose gestational ages were confirmed by ultrasonographic techniques in early gestation with the cooperation of 21 major medical centers throughout Japan. The 10th, 50th, and 90th percentiles for each body size parameter according to sex are presented. For birth weight, parity was also considered.

The twins’ standards we adopted were the recently reported birth weight standards in Japan.^14^ These standards were calculated based on birth records in the vital statistics from 1988 through 1992, which included about 64,000 live birth twin individuals. The 10th, 50th, and 90th percentiles of birth weight by gestational ages from 24 to 41 weeks according to sex and parity are presented. No reliable standards for length, chest circumference, and head circumference were available for twins in Japan.

## RESULTS

The results of stepwise regression analysis showed that the contribution of gestational age was the strongest (R^2^=0.31, p=0.0001 for birth weight, R^2^=0.30, p=0.0001 for length, R^2^=0.25, p=0.0001 for chest circumference, and R^2^=0.18, p=0.0001 for head circumference). Sex and parity also contributed to body size parameters, albeit slightly. The effect of parity was second largest (p=0.0001) influence on birth weight, though the effect itself was not so large (R^2^=0.02).

Body size parameters according to sex and parity are shown in Table 2. Irrespective of sex, body size parameters of twins became larger for multipara than for primipara. This tendency was clearly seen in relation to birth weight.

**Table 2.** Birth weight, length, chest circumference, and head circumference of twins according to parity.

	parity	male			female ^a^		
	
		n	mean	standard deviation	n	mean	standard deviation
weight (kg)	0	514	2.45	0.45	575	2.39	0.43
	1	384	2.56	0.44	417	2.50	0.39
	2-3	95	2.63	0.53	127	2.60	0.47

length (cm)	0	471	46.9	2.6	540	46.2	2.6
	1	368	47.1	2.5	393	46.8	2.5
	2-3	90	47.6	2.6	124	46.9	2.4

chest circumference (cm)	0	436	29.6	2.2	515	29.5	2.2
	1	339	30.0	2.2	360	29.9	2.2
	2-3	81	30.3	2.5	106	30.2	2.4

head circumference (cm)	0	437	32.3	1.7	515	32.0	1.5
	1	339	32.6	1.6	361	32.1	1.6
	2-3	81	32.6	2.0	106	32.3	1.8

a: Parity was unknown as to one set of female twin pairs.

From analyzing the body size parameter data in terms of missing data, it became obvious that birth weight and length were measured more often than chest circumference and head circumference. The number of missing values for birth weight and length were 8 (0.4%) and 134 (6.3%) respectively, whereas the number of missing values for chest circumference and head circumference were 283 (13.3%) and 281 (13.2%), respectively. Two possible reasons were (1) the avoidance of troublesome work, or (2) an emergency circumstance, in which there was no time to measure parameters other than weight and/or length. To confirm the latter possibility, birth weight or birth length was compared according to the missing data condition; the results are shown in Table 3. Indeed, a significantly light birth weight was observed when the other three items were not measured. No significant difference of length was observed when chest or head circumference were not measured. Thus, birth weight could be underestimated if standards were developed using only subjects with a full set of measurements.

**Table 3.** Birth weight and length according to several conditions of missing data.

	male					

	length		chest circumference		head circumference	
		
	data exist	data not exist	data exist	data not exist	data exist	data not exist
weight (kg)	2.54 (n=929)	2.00 (n=64)***	2.55 (n=856)	2.25 (n=137)***	2.55 (n=857)	2.25 (n=136)***
length (cm)	-	-	47.0 (n=854)	46.9 (n=75)	47.0 (n=855)	47.0 (n=74)

	female					

	length		chest circumference		head circumference	
		
	data exist	data not exist	data exist	data not exist	data exist	data not exist

weight (kg)	2.48 (n=1056)	2.03 (n=65)***	2.49 (n=980)	2.21 (n=141)***	2.49 (n=981)	2.22 (n=140)***
length (cm)	-	-	46.5 (n=979)	46.1 (n=80)	46.5 (n=980)	46.1 (n=79)

*** : p<0.001. data exist vs data not exist.

Using subjects that had data on gestational age and all four parameters (n=841 for males and n=962 for females), we calculated correlation coefficients of body size parameters. Gestational age was correlated with weight (r=0.576 for males, r=0.538 for females), length (r=0.555 for males, r=0.514 for females), chest circumference (r=0.513 for males, r=0.492 for females), and head circumference (r=0.462 for males, r=0.418 for females), in this order for both sexes; the results were in accordance with the regression analyses. Birth weight showed high correlations with length (r=0.801 for males, r=0.770 for females) and chest circumference (r=0.828 for males, r=0.810 for females), and a slightly lower correlation with head circumference (r=0.691 for males, r=0.688 for females).

**Figure 1.**
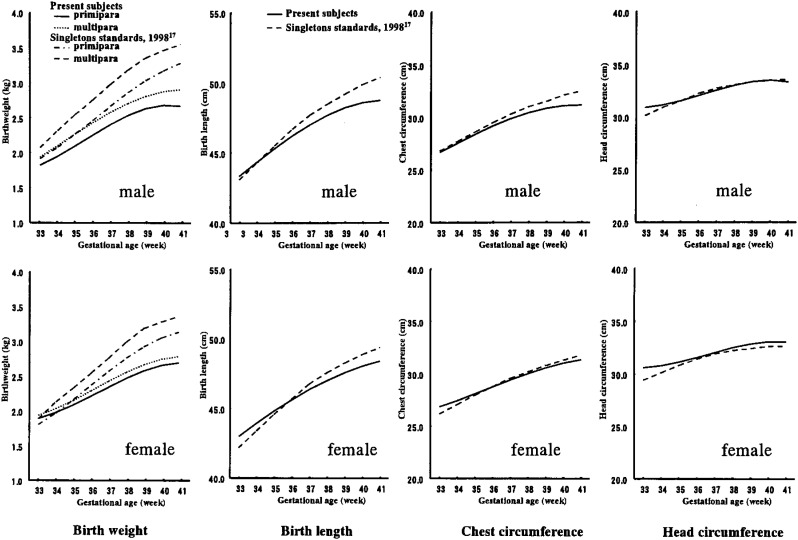
50th percentiles for twins compared with singletons standards.

Reference growth standards data are presented in Table 4. Birth weight references were calculated separately for primipara and multipara according to the standards for singletons^17^ and twins,^14^ considering the effect of parity. As the sample size below 32 weeks was small, smoothing was performed only for 33-41 weeks gestation. Smoothed 50th percentile curves are compared with singleton norms (Figure 1).^17^ Compared with singletons, the weight deficit started from 33 weeks’ gestation and became more marked by the week. Not so markedly as weight, the size deficit according to gestational week was observed primarily in regards to length. No deficit was observed for chest or head circumference.

**Table 4.** Birth weight, length, chest circumference, and head circumference of twins by gestational age.

	gestational age (week)	male						female					
	
		n	mean	SD	percentiles			n	mean	SD	percentiles		
	
					10th	50th	90th				10th	50th	90th
weight (kg) primipara	27-32	20	1.694	0.163	1.342	1.734	2.018	23	1.574	0.236	1.402	1.853	2.009
	33	15	1.885	0.250	1.456	1.826	2.190	11	1.838	0.307	1.537	1.901	2.099
	34	19	1.872	0.273	1.585	1.954	2.365	19	2.109	0.223	1.659	1.986	2.235
	35	46	2.080	0.326	1.722	2.102	2.537	59	2.046	0.298	1.769	2.097	2.401
	36	48	2.314	0.280	1.861	2.259	2.704	32	2.152	0.240	1.869	2.222	2.580
	37	58	2.466	0.347	1.994	2.409	2.860	84	2.422	0.307	1.959	2.352	2.756
	38	117	2.544	0.323	2.113	2.539	3.002	122	2.473	0.363	2.039	2.475	2.911
	39	87	2.673	0.408	2.212	2.635	3.126	119	2.583	0.419	2.110	2.581	3.028
	40	62	2.630	0.389	2.284	2.684	3.226	72	2.681	0.358	2.173	2.659	3.091
	41	32	2.770	0.493	2.321	2.671	3.300	16	2.647	0.283	2.228	2.697	3.083

weight (kg) multipara	27-32	9	1.672	0.212	1.489	1.781	2.094	7	1.619	0.181	1.653	1.891	2.294
	33	13	1.932	0.229	1.606	1.941	2.258	5	2.129	0.198	1.662	1.948	2.377
	34	21	2.060	0.366	1.731	2.108	2.442	15	2.108	0.262	1.713	2.042	2.489
	35	41	2.313	0.296	1.861	2.275	2.636	69	2.184	0.380	1.794	2.162	2.621
	36	44	2.397	0.359	1.989	2.435	2.829	32	2.273	0.412	1.896	2.296	2.765
	37	93	2.525	0.296	2.112	2.582	3.009	81	2.517	0.287	2.008	2.433	2.912
	38	106	2.699	0.420	2.224	2.709	3.167	133	2.576	0.319	2.121	2.562	3.055
	39	70	2.837	0.370	2.320	2.810	3.291	110	2.664	0.340	2.225	2.671	3.184
	40	58	2.882	0.404	2.397	2.877	3.371	64	2.757	0.376	2.308	2.749	3.291
	41	11	2.880	0.324	2.448	2.905	3.396	15	3.003	0.516	2.361	2.784	3.368

length (cm)	27-32	14	40.8	1.9	38.0	40.5	43.0	18	41.4	2.3	37.5	41.3	45.0
	33	22	43.3	2.5	39.9	43.3	46.1	16	42.8	2.0	40.5	43.0	45.0
	34	33	43.9	2.2	41.1	44.4	47.0	33	44.1	1.6	41.0	44.0	46.1
	35	80	45.3	2.3	42.3	45.3	47.9	114	44.7	2.3	41.7	44.8	47.2
	36	87	46.1	2.1	43.5	46.2	48.7	57	44.8	2.4	42.5	45.6	48.1
	37	142	47.0	1.7	44.5	47.0	49.4	160	46.6	2.0	43.3	46.4	48.8
	38	219	47.7	2.1	45.3	47.7	50.0	250	46.7	2.3	44.1	47.0	49.5
	39	150	48.4	1.8	45.8	48.2	50.4	226	47.4	2.1	44.6	47.6	49.9
	40	119	48.0	2.3	45.9	48.6	50.6	129	48.0	2.0	45.0	48.1	50.2
	41	40	48.3	2.3	45.5	48.7	50.5	31	48.2	2.3	45.1	48.4	50.4

chest circumference (cm)	27-32	10	25.2	1.7	23.3	25.1	27.5	12	25.3	1.5	23.5	25.3	27.0
	33	20	26.6	1.6	24.2	26.7	28.8	12	26.9	1.8	24.1	26.9	28.8
	34	30	27.3	1.7	25.2	27.5	29.9	28	27.2	1.3	24.9	27.5	29.5
	35	75	28.2	2.0	26.0	28.4	30.7	102	27.9	2.3	25.6	28.1	30.3
	36	83	29.4	1.8	26.7	29.2	31.4	57	28.8	2.2	26.4	28.8	31.1
	37	132	29.7	1.8	27.3	29.9	31.9	148	29.7	1.9	27.0	29.4	31.8
	38	203	30.1	2.1	27.8	30.4	32.4	233	30.0	1.9	27.6	30.0	32.5
	39	143	30.9	1.8	28.2	30.8	32.9	213	30.4	2.0	28.1	30.6	33.0
	40	106	30.7	2.0	28.5	31.1	33.4	128	30.8	1.8	28.5	31.0	33.3
	41	34	31.3	2.2	28.7	31.1	34.1	28	31.3	1.8	28.7	31.4	33.3

head circumference (cm)	27-32	10	28.7	1.2	27.0	28.7	30.3	12	29.1	1.4	27.5	28.8	31.0
	33	20	30.6	1.3	28.7	30.9	32.2	12	30.4	1.1	29.2	30.6	31.7
	34	30	30.6	1.5	29.1	31.1	32.7	28	30.9	1.2	28.8	30.8	32.5
	35	76	31.4	1.6	29.6	31.5	33.1	103	30.9	1.5	29.0	31.1	33.0
	36	84	32.0	1.5	30.2	32.0	33.6	57	31.4	2.1	29.4	31.5	33.4
	37	131	32.5	1.6	30.7	32.5	34.0	148	32.0	1.3	30.0	32.0	33.6
	38	203	32.6	1.5	31.2	33.0	34.4	232	32.3	1.4	30.6	32.5	33.8
	39	143	33.1	1.4	31.5	33.3	34.7	215	32.5	1.4	31.0	32.8	34.1
	40	106	33.2	1.5	31.6	33.4	34.9	127	32.7	1.4	31.0	33.0	34.5
	41	34	33.3	1.5	31.4	33.3	35.1	28	32.8	1.8	30.5	33.0	35.0

SD: standard deviation

**Figure 2.**
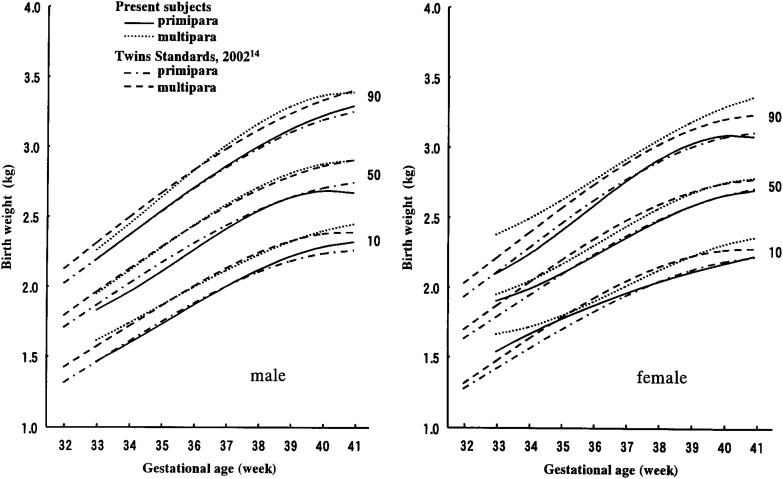
Birth weight percentiles for twins compared with twins standards.

A comparison with the birth weight standards for twins^14^ is shown in Figure 2. Figures for the 50th percentile of the present curves mostly landed consistently near the norms irrespective of parity and sex.

## DISCUSSION

Gestational age proved the strongest contributing factor to body size parameters at birth. Other significant factors were sex and parity. Males were heavier and larger than females. The results of stepwise regression analysis and the data in Table 2 indicated that the effect of parity on body size parameters, especially on birth weight, was independent of gestational age. An effect of zygosity on birth weight and length was also observed, but the effect was not as large (data not shown). These results were in accordance with previous reports.^19^^-^^21^ A contribution of chorionisity to birth weight has also been pointed out.^13^^,^^22^ The present data did not contain information on chorionisity.

The sample size of this study is not as large as population-based twin studies often performed in western countries, especially for lower gestational weeks. In Japan, it is considerably difficult to simultaneously obtain obstetrical records and body size parameters at birth on a large number of twins, since there is no population-based twin registry. Previous norms of twin birth weights reported in Japan have been mainly based on hospital data with several hundred twin pairs; these have considerably underestimated birth weight.^8^ In Japan, the birth record in the vital statistics is available for birth weight information,^1^^,^^2^^,^^14^ calculated by 100g intervals. From 1996 on, birth length was added to the vital statistics and birth weight was recorded to an accuracy of 1g.^14^ Birth weight or length norms of twins using this new data have not yet been presented. Other body size parameters at birth cannot be obtained through the birth record at present. In contrast, the present data was based on the records in the “Maternal and Child Health Handbook,” which was recorded by obstetricians with considerable accuracy, though the methods of measurements may vary and thus the accuracy of data could not be ascertained directly.

The present subjects were normally developed twins. Therefore, growth norms seemed to be larger than the general twin population. Compared with hospital data,^8^ which includes data from a disproportionate number of high-risk twins and is known to underestimate body size parameters, however, the present percentiles should more closely reflect the characteristics of the general twin population. As shown in Figure 2, the present results of 50th percentile curves consistently fell near the norms of primipara and multipara of birth weight norms of twins using the vital statistics in Japan.^14^ This fact supports our claim that present data more closely reflects real body size parameters of the general twin population than hospital data.

Birth weight in twins was well correlated with birth length and chest circumference, although the correlation between birth weight and head circumference was slightly lower, suggesting a need for growth standards on head circumference. Birth weight was significantly lighter when chest or head circumference could not be measured. To ascertain the possible cause of the absence of certain data, we analyzed the frequencies of neonatal asphyxia according to the missing data condition. In cases in which length, chest circumference, and head circumference were not measured, the frequency of neonatal asphyxia was 14.2% (19/134), 10.6% (30/283), and 10.7% (30/281), respectively. In cases in length, chest circumference, and head circumference were measured, the frequency of neonatal asphyxia was 5.1% (102/1988), 5.0% (91/1839), and 4.9% (91/1841), respectively. These findings strongly suggested that the reason for missing data was an emergency situation; these subjects may include a disproportionate number of unhealthy or at-risk infants. Conversely, chest or head circumference may only be measured for relatively healthy neonates. For these two parameters, it seems important to bear the possibility of positive selection bias in mind.

As shown in Figure 1, the growth curves of birth weight for twins were considerably different from singletons, a phenomenon which has been pointed out many times. The present study confirms this point using a Japanese sample.

As to length, a slight deficit in twins was observed. About a 1cm deficit was observed at 41 weeks for both sexes. Little has been reported^6^^,^^9^ as to this parameter; moreover, it has been pointed out^12^ that length is unreliable, with many different measurement techniques. Nevertheless, the results of this study were in accordance with previous reports. The length of twins became larger with gestational age, and males were, on average, larger.

Chest and head circumference were nearly the same as or even larger than those of singletons, partly suggesting the positive selection bias mentioned above. According to Bucker and Green,^12^ who analyzed over 5,300 head circumferences of twins, differences in head circumferences between singletons and twins are only evident with gestations longer than 35 weeks, and from 37 weeks’ gestation onwards, the mean head circumference of singletons exceeded that of twins by only 5 mm. The deficit of head circumference in twins, though it exists, is certainly not so large as birth weight.

It is said that previous population-based and large hospital-based investigations do not agree with regard to the gestational age at which singleton and twin growth curves start to differ.^3^ Besides, the difference between the results of most of the studies may partly result from the differences in study populations. Taking all these things in consideration, it seems very difficult to clarify the growth difference between singletons and twins meaningfully using the present sample.

It is important to recognize that often-reported body size standards by gestational age are cross-sectional and essentially different from real intrauterine growth or longitudinal measurements by ultrasound examination.^23^^-^^25^ Each method has its merits and demerits.

Birth years of the present sample were distributed roughly over twenty years. It has been recommended that birth weight norms should be updated every 5-10 years,^10^ because secular trends have been observed. In this study, secular trends of body size were not considered, partly because no strong birth year effect was observed by regression analysis.

In interpreting the results of the present study, the following selection biases should be considered. First, both twins are alive and have shown no marked growth disturbance through age 11 or 12. Second, the subjects are only in the Tokyo Metropolitan aria. Third, the subjects are all applicants to an entrance examination for a university-affiliated school. The direct effect of these selection biases is difficult to specify. The selection bias should be much lower in the population-based study using the vital statistics, which theoretically contain the data of most live-born twins of the year. We think the present data may offer reference birth size parameters for normal development through infancy, or the upper limit of intrauterine growth norms.

Though there were several limitations in the present results, some of the features related to body size parameters at birth in twins were made clear. As shown in Figure 2, the present results were consistently similar to the only report of birth weight norms of twins that uses vital statistics in Japan. This corroboration independently supports the claim that both of the studies more closely reflect real body size parameters of the general twin population than hospital data. It was concluded that growth standards for twins are essential for understanding and evaluating intrauterine growth of twins. Moreover, both our report and that based on vital statistics are critical for gauging the health and viability of twins in Japan.
